# Synthesis, crystal structure and spectroscopic and Hirshfeld surface analysis of 4-hy­droxy-3-meth­oxy-5-nitro­benzaldehyde

**DOI:** 10.1107/S2056989020000225

**Published:** 2020-01-21

**Authors:** Vitomir Vusak, Darko Vusak, Kresimir Molcanov, Mestrovic Ernest

**Affiliations:** a PLIVA Croatia Ltd., Prilaz baruna Filipovića 29, HR-10000 Zagreb, Croatia; b Department of Chemistry, Faculty of Science, University of Zagreb, Horvatovac, 102a, HR-10000 Zagreb, Croatia; c Department of Physical Chemistry, Ruđer Bošković Institute, Bijenička cesta 54, HR-10000 Zagreb, Croatia

**Keywords:** crystal structure, entacapone, hydrogen bonding, Hirshfeld surface analysis

## Abstract

The title compound, C_8_H_7_NO_5_, is planar with an r.m.s. deviation for all non-hydrogen atoms of 0.018 Å. An intra­molecular O—H⋯O hydrogen bond involving the adjacent hy­droxy and nitro groups forms an *S*(6) ring motif. In the crystal, mol­ecules are linked by O—H⋯O hydrogen bonds, forming chains propagating along the *b*-axis direction. The chains are linked by C—H⋯O hydrogen bonds, forming layers parallel to the *bc* plane. The layers are linked by a further C—H⋯O hydrogen bond, forming slabs, which are linked by C=O⋯π inter­actions, forming a three-dimensional supra­molecular structure. Hirshfeld surface analysis was used to investigate inter­molecular inter­actions in the solid state. The mol­ecule was also characterized spectroscopically and its thermal stability investigated by differential scanning calorimetry and by thermogravimetric analysis.

## Abstract 2

The title mol­ecule is planar with an r.m.s. deviation for all non-hydrogen atoms of 0.018 Å. An intra­molecular O1—H1⋯O5 hydrogen bond involving the adjacent hydroxyl and nitro groups closes an *S*(6) ring motif.

## Chemical context   

The title compound is a key starting material in the preparation of entacapone (Srikanth *et al.*, 2012 ▸; Mantegazza *et al.*, 2008 ▸; Chinnapillai Rajendiran *et al.*, 2007 ▸; Deshpande *et al.*, 2010 ▸). Entacapone, (*E*)-2-cyano-*N,N*-diethyl-3-(3,4-dihy­droxy-5-nitro­phen­yl)propenamide (**II**), whose crystal structure has been reported by Leppänen *et al.* (2001 ▸), is a selective and reversible catechol-*O*-methyl­transferase inhib­itor used in the treatment of Parkinson’s disease in combin­ation with levodopa and carbidopa (Najib, 2001 ▸; Pahwa & Lyons, 2009 ▸). Entacapone (**II**), prevents metabolism and inactivation of levodopa and carbidopa, which allows better bio-availability of these compounds. Several synthetic routes for the synthesis of entacapone have been reported (Bartra Sanmarti *et al.*, 2008 ▸; Harisha *et al.*, 2015 ▸; Jasti *et al.*, 2005 ▸; Cziáky, 2006 ▸); however, only a few inter­mediates/starting materials have been characterized crystallographically (Keng *et al.*, 2011 ▸; Babu *et al.*, 2009 ▸; Vladimirova *et al.*, 2016 ▸). Knowledge of the crystal structure is beneficial for understanding the properties of the starting materials as well as being the gold standard for the identification of starting materials. Recently, we have synthesized and studied the influence of different entacapone-related compounds on the crystallization of the final forms of entacapone. As part of this work, the title compound, 4-hy­droxy-3-meth­oxy-5-nitro­benzaldehyde (**I**), was synthesized and its spectroscopic and structural features were studied. There are two reasons for this study, one is connected with the utilization of crystal structures in the identification of materials in the solid state, and the other is to build a library of structurally related compounds of entacapone that will be utilized in a future crystallization study.
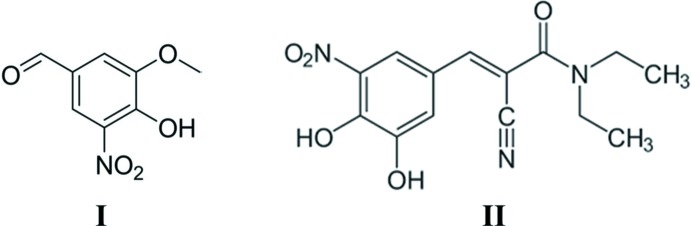


## Structural commentary   

**Figure 1:**
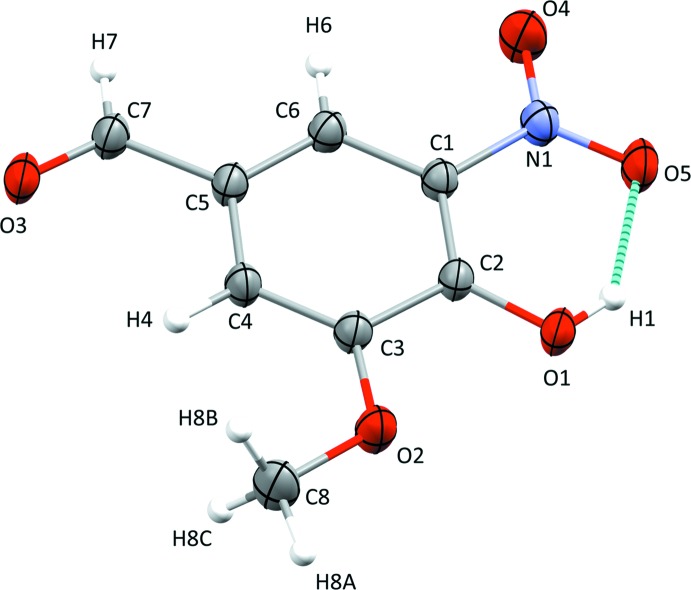
The mol­ecular structure of compound **I**, with atom labelling. Displacement ellipsoids are drawn at the 50% probability level. The intra­molecular O1—H1⋯O5 hydrogen bond is shown as a dashed line (Table 1 ▸).

The mol­ecular structure of the title compound **I** is illustrated in Fig. 1 ▸. The intra­molecular O1—H1⋯O5 hydrogen bond (Table 1 ▸), involving the adjacent hydroxyl and nitro groups, forms an *S*(6) ring motif. The mol­ecule is planar (r.m.s. deviation for all non-hydrogen atoms is 0.018 Å) with the maximum deviation from the mean plane being 0.038 (1) Å for atom O5. The bonds lengths and bond angles are close to those found for similar structures (see §4. *Database survey*).

## Supra­molecular features   

**Figure 2:**
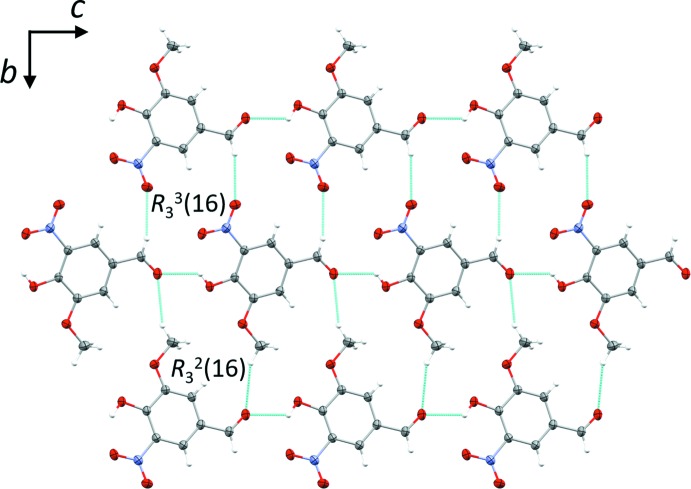
A partial view along the *a* axis of the crystal packing of compound **I**, illustrating the two different ring motifs. The hydrogen bonds (Table 1 ▸) are shown as dashed lines.

**Figure 3:**
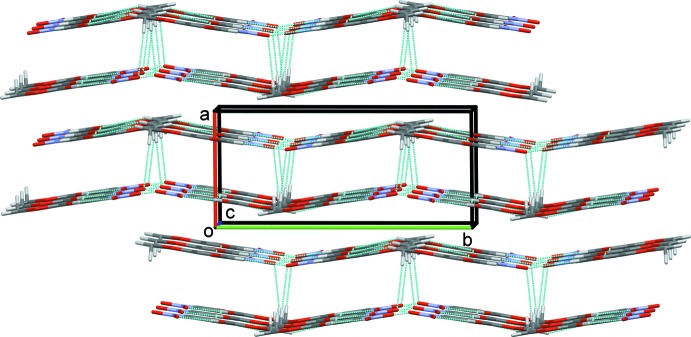
A view along the *c* axis of the crystal packing of compound **I**. The hydrogen bonds (Table 1 ▸) are shown as dashed lines. For clarity, only the H atoms involved in hydrogen bonding have been included.

**Figure 4:**
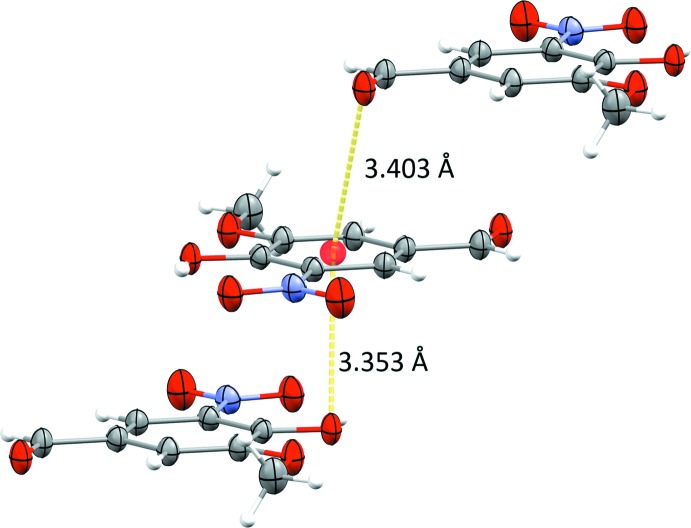
Short C—O_hy­droxy_⋯π and C=O_aldehyde_⋯π contacts.

In the crystal of compound **I**, mol­ecules are linked by inter­molecular bifurcated hydrogen bonds involving the hydroxyl group. Details of the hydrogen bonding together with the symmetry codes are given in Table 1 ▸. The O1—H1⋯O3^i^_aldehyde_ hydrogen bonds [2.6989 (12) Å] link the mol­ecules into chains with a *C*(8) motif. Each oxygen atom is involved in one or more inter­molecular hydrogen bonds, except for the O5_nitro_ atom, which is involved only in the intra­molecular hydrogen bond. Each mol­ecule is connected by six adjacent mol­ecules through strong O1—H1⋯O3_aldehyde_ and weak C7—H7⋯O4^ii^_nitro_ and C8—H8*A*⋯O3^iii^_aldehyde_ hydrogen bonds, forming undulating layers parallel to the *bc* plane, enclosing two type of ring motifs – 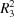
(16) and 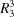
(16) (Fig. 2 ▸). The layers are linked by a further C—H⋯O hydrogen bond, C8—H8*B*⋯O4^iv^_nitro_, forming slabs (Fig. 3 ▸). Moreover, C7=O3⋯*π* [oxygen–centroid distance = 3.4028 (12) Å] and C2—O1⋯*π* [3.353 **(su?)** Å] close contacts (Fig. 4 ▸) are present, linking the slabs to form a three-dimensional supra­molecular structure.

## Database survey   

A search of the Cambridge Structural Database (CSD, Version V5.41, last update November 2019; Groom *et al.*, 2016 ▸), for crystal structures containing a nitro group on the benzene ring, oxygen atoms bonded on carbon position 2 and 3, and the –C=O group located on position 5, gave three hits, out of which only one entry contained the title mol­ecule, *viz*. a tin complex of the 4-hy­droxy-3-meth­oxy-5-nitro­benzaldehyde with a deprotonated hydroxyl group and benzyl anions (CSD refcode EREWII; Keng *et al.*, 2011 ▸). The other two entries do not contain an aldehyde group, but a methyl­keto (MUCDOE; Babu *et al.*, 2009 ▸) and carb­oxy­lic group (TAFSAX; Vladimirova *et al.*, 2016 ▸) instead.

A second search of the CSD for a nitro group on a benzene ring, OH groups on carbon atoms 2 and 3, and a carbon atom on position 5 gave eight hits for seven structures. These include the structure of entacapone **II** (OFAZUQ; Leppanen *et al.*, 2001 ▸), and four of its acyl esters, *viz*. (*E*)-2-cyano-3-(3,4-dihy­droxy-5-nitro­phen­yl)-*N*,*N*-di­ethyl­prop-2-enamide 1,3-di­methyl-3,7-di­hydro-1*H*-purine-2,6-dione monohydrate (XIPNOC; Bommaka *et al.*, 2018 ▸), (*E*)-2-cyano-3-(3,4-dihy­droxy-5-nitro­phen­yl)-*N*,*N*-di­ethyl­prop-2-enamide pyridine-4-carboxamide (XIPNUI; Bommaka *et al.*, 2018 ▸), (*E*)-2-cyano-3-(3,4-dihy­droxy-5-nitro­phen­yl)-*N*,*N*-di­ethyl­prop-2-enamide pyrazine-2-carboxamide (XIPPAQ; Bommaka *et al.*, 2018 ▸), and (*E*)-2-cyano-3-(3,4-dihy­droxy-5-nitro­phen­yl)-*N*,*N*-di­ethyl­prop-2-enamide acetamide (XIPPEU; Bommaka *et al.*, 2018 ▸). These four compounds were prepared by solvent-assisted grinding, and the study was aimed at improving the aqueous solubility, diffusion permeability, and co-crystal stability of entacapone.

## Hirshfeld surface analysis   

**Figure 5:**
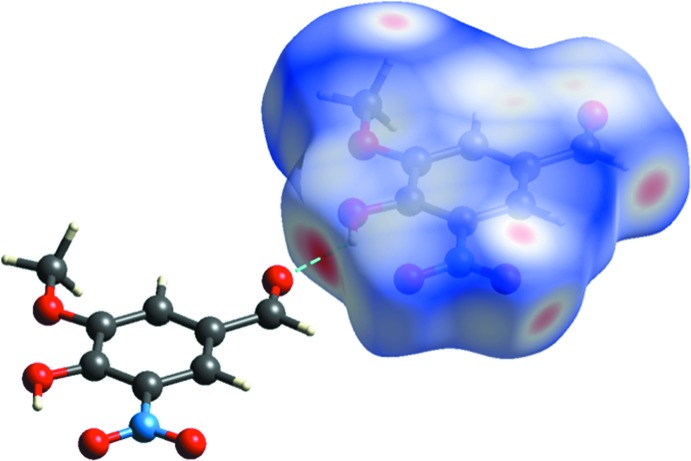
A view of the Hirshfeld surface of compound **I**, mapped over *d*_norm_ in the colour range −0.448 to 1.186 a.u.. Red areas show inter­molecular contacts shorter than the sum of the van der Waals radii of the atoms. The shortest inter­molecular O—H⋯O hydrogen bond is also shown.

Inter­molecular inter­actions in the crystal of compound **I** were further investigated by the Hirshfeld surfaces. Calculations were performed using *CrystalExplorer17* (Turner *et al.*, 2017 ▸). The *d*_norm_ values were mapped onto the Hirshfeld surface over the whole mol­ecule (Fig. 5 ▸). Red areas represent contacts of the atoms shorter then the sum of the van der Waals radii, such as hydrogen bonds, or C=O⋯*π* contacts, whereas blue areas represent contacts between atoms longer then the sum of the van der Waals radii. White areas represent contacts equal to the sum of the van der Waals radii.

**Figure 6:**
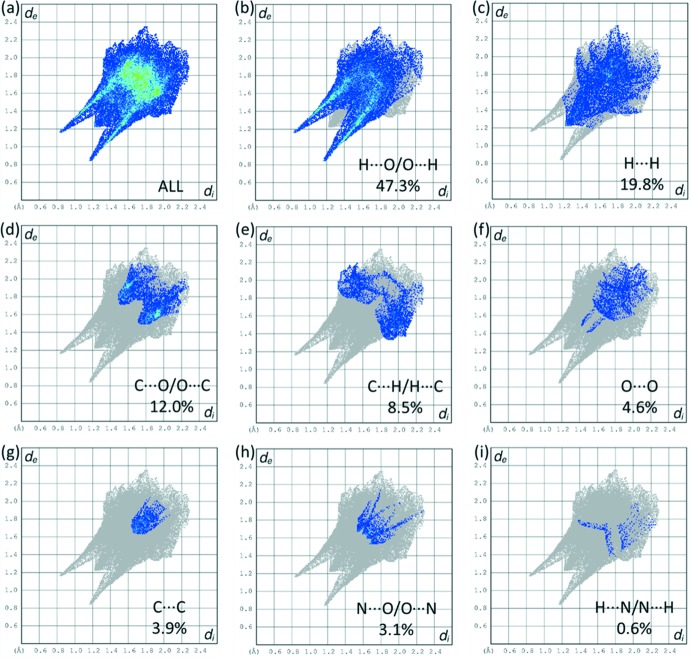
(*a*) Two-dimensional fingerprint plot for compound **I**, and the fingerprint plots delineated into (*b*) H⋯O/O⋯H (47.3%), (*c*) H⋯H (19.8%), (*d*) C⋯O/O⋯C (12.0%), (*e*) C⋯H/H⋯C (8.5%), (*f*) O⋯O (4.6%), (*g*) C⋯C (3.9%), (*h*) N⋯O/O⋯N (3.1%), (*i*) H⋯N/N⋯H (0.6%).

The two-dimensional fingerprint plots show inter­molecular contacts and distances between atoms (Fig. 6 ▸). The most abundant contacts are between oxygen and hydrogen atoms, comprising almost half of the Hirshfeld surface area (47.3%). This finding is not surprising having in mind the number of oxygen atoms located on the edges of the mol­ecule with respect its size, each of them is involved in one or more hydrogen bonds. Also, because of the large number of oxygen atoms and C=O⋯*π* contacts, there is a high proportion of C⋯O/O⋯C contacts, which comprise 12.0% of the surface area. Since there is no stacking of the aromatic rings, only 3.9% of the surface derives from C⋯C contacts.

**Figure 7:**
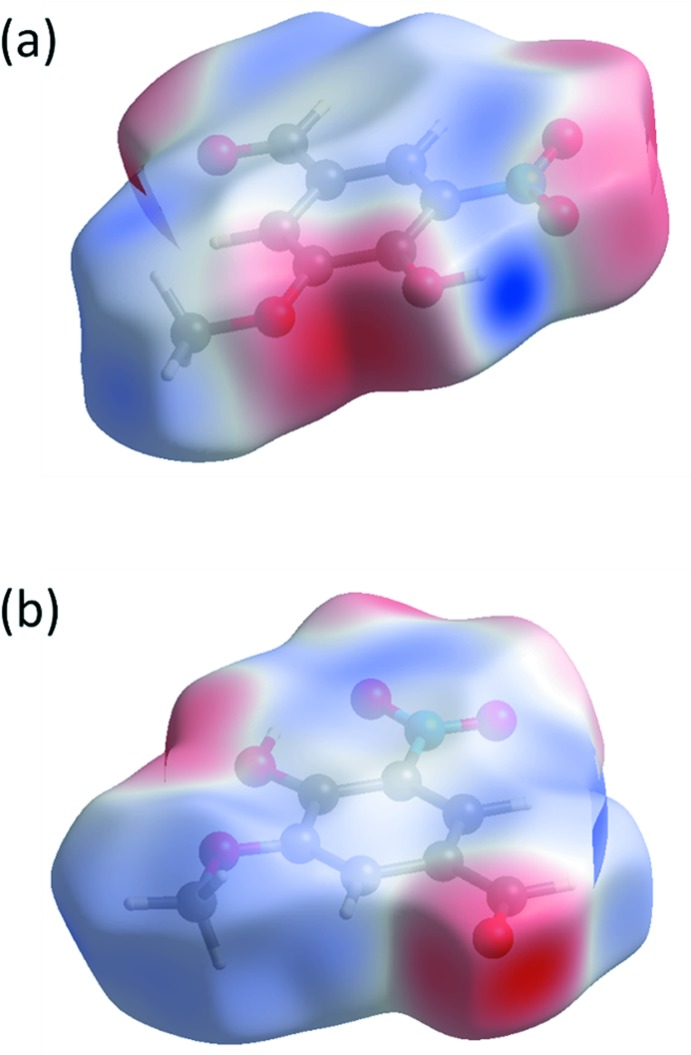
Calculated electrostatic potentials over the Hirshfeld surface of compound **I**. Electrostatic potential was mapped in the energy range −0.0923 to 0.1232 a.u.. The blue area around the hydroxyl oxygen atom in (*a*) represents the most positive part, while the red area around the carbonyl oxygen atom in (*b*) represents the most negative part of the mol­ecule.

Electrostatic potentials were calculated using *TONTO* with a 3-21G basis set at the Hartree–Fock level of theory and were mapped on the Hirshfeld surface (Fig. 7 ▸) in the energy range between −0.0923 and 0.1232 a.u.. The most positive region is around the hydroxyl hydrogen atom (Fig. 7 ▸ *a*), while the most negative region is around the carbonyl oxygen atom (Fig. 7 ▸ *b*). Those two atoms are involved in the shortest inter­molecular hydrogen bond in the crystal structure (O1—H1⋯O3^i^), where O1⋯O3^i^ = 2.6989 (12) Å; see Table 1 ▸.

## Synthesis and crystallization   

4-Hy­droxy-3-meth­oxy­benzaldehyde (20 g; 131.4 mmol) was dissolved in acetic acid (200 ml) and the solution was cooled to 283–288 K and 65% HNO_3_ (10.5 ml) was added dropwise over 30 min. The reaction mixture was stirred for 30 min at 283–288 K and 30 min at 293–298 K. The suspension was then filtered and the crystals obtained were washed with water (3 × 20 ml). The crystals were dried in a vacuum dryer (10 mbar, 313 K, 16 h) to obtain pure yellow compound **I** (yield 20.28 g; 78.3%). Yellow block-like crystals, suitable for X-ray diffraction analysis, were obtained by slow evaporation of a solution in acetone after 10 d at room temperature.

**Figure 8:**
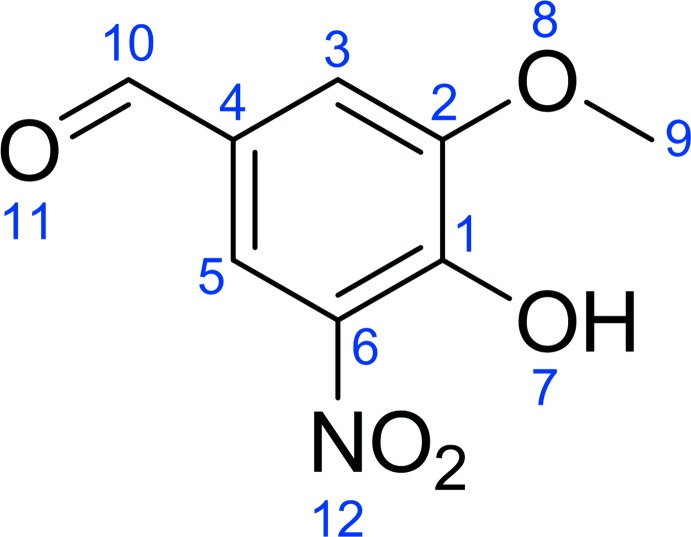
Structure of compound **I** in relation to the NMR data in Tables 2 ▸ and 3 ▸.

**Figure 9:**
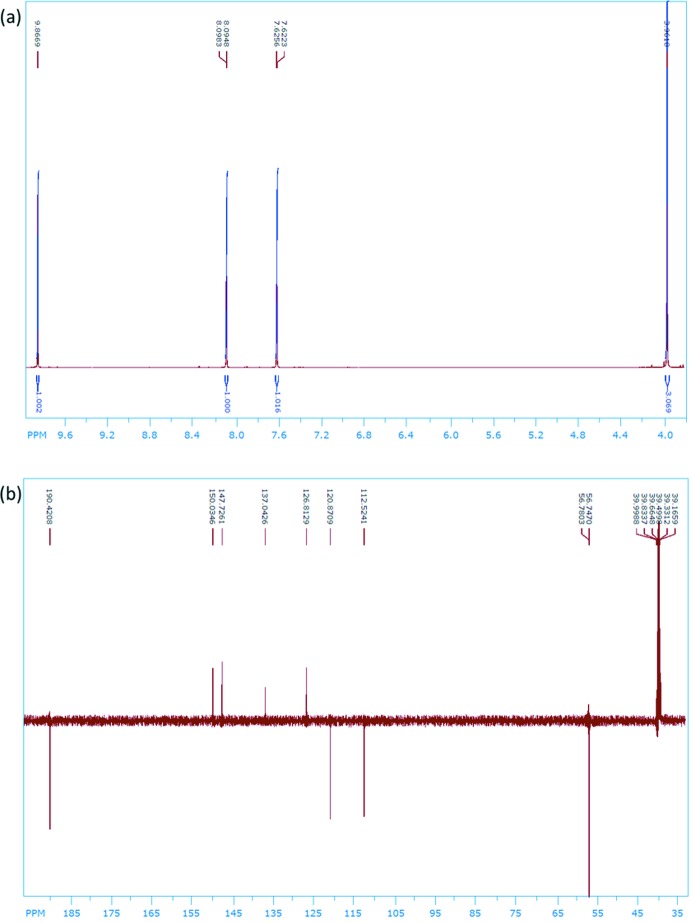
^1^H NMR spectra of compound **I** (CD_3_OD); (*b*) ^13^C NMR spectra of compound **I** (CD_3_OD)

*Spectroscopic analysis*:

The structure of compound **I** (Fig. 8 ▸) was confirmed by ^1^H and ^13^C NMR, recorded on a Bruker Avance DRX 500 at 500.1 MHz (^1^H) and 125.8 MHz (^13^C) in CD_3_OD (Fig. 9 ▸ *a* and 9*b*, respectively); see Tables 2 ▸ and 3 ▸ for further details.

## Thermal analysis   

**Figure 10:**
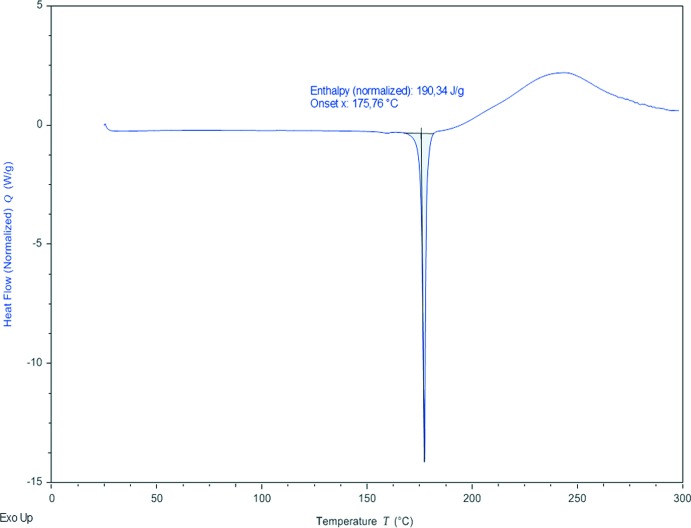
DSC curve of compound **I**.

**Figure 11:**
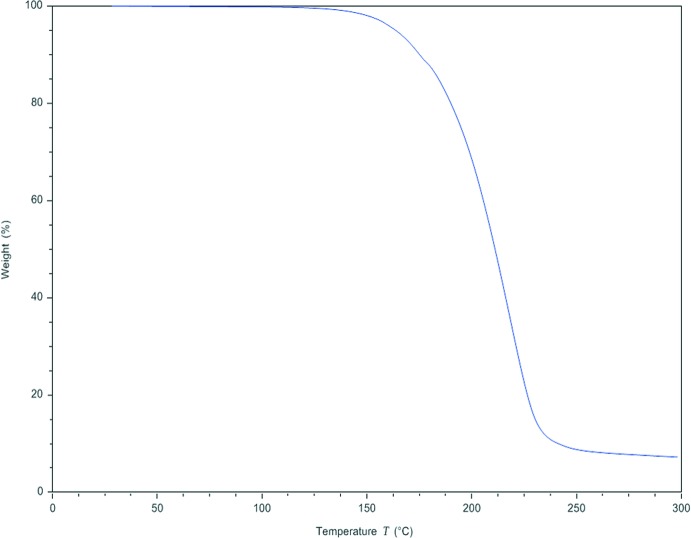
TG curve of compound **I**.

The thermal stability of compound **I** was investigated in the solid state by differential scanning calorimetry (DSC) and by thermogravimetric analysis (TGA). DSC analysis was performed on a TA Instruments Discovery DSC in a closed aluminium pan (40 µL) under nitro­gen flow (50 ml min^−1^) and a heating rate of 10°C min^−1^ in the temperature range 25–300 °C (Fig. 10 ▸). Thermogravimetric analysis was performed on a TA Instruments Discovery TG in a closed aluminium pan (40 µL) under nitro­gen flow (50 ml min^−1^) and a heating rate of 10°C min^−1^ in the temperature range 25–300°C (Fig. 11 ▸). DSC analysis shows one endotherm at about 176°C that corresponds to the melting point of the title compound. Thermogravimetric analysis does not show any weight loss during heating up to 140°C where a change in mass can be observed that can be attributed to the thermal decomposition of the sample.

## IR spectroscopy   

**Figure 12:**
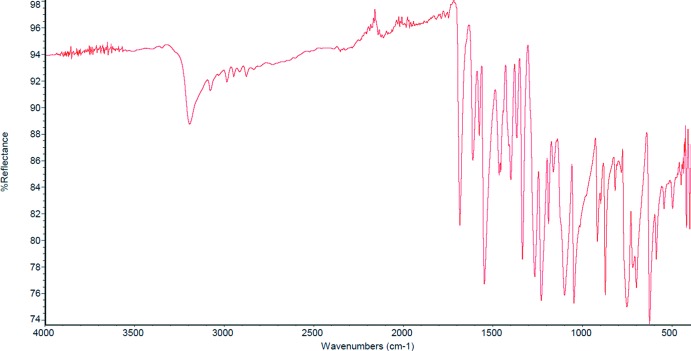
IR spectra (ATR) of compound **I**.

The IR spectrum (Fig. 12 ▸) of compound **I** was recorded on a Thermo Scientific Nicolet instrument by ATR (attenuated total reflectance) technique. It shows a broad band at about 3200 cm^−1^, which corresponds to the O—H stretching vibrations. Strong stretching vibrations of C=O (aldehyde) and C—O (aromatic ether) appear at 1683 and 1266 cm^−1^, respectively. Bands corresponding to N—O asymmetric and symmetric stretching modes can be found at 1547 and 1366 cm^−1^, respectively. Characteristic weak overtones of the aromatic ring can be seen at 1800–1700 cm^−1^.

## Refinement details   

Crystal data, data collection and structure refinement details are summarized in Table 4 ▸. Hydrogen atoms were located in a difference-Fourier map and refined as riding on their parent atom: O—H = 0.82 Å, C—H = 0.93-0.96 Å, with *U*_iso_(H) = 1.5*U*_eq_(O) and 1.2*U*_eq_(C) for other H atoms.

## Supplementary Material

Crystal structure: contains datablock(s) global, I. DOI: 10.1107/S2056989020000225/su5534sup1.cif

Structure factors: contains datablock(s) I. DOI: 10.1107/S2056989020000225/su5534Isup2.hkl

Supporting information file. DOI: 10.1107/S2056989020000225/su5534Isup3.cml

CCDC reference: 1957893

Additional supporting information:  crystallographic information; 3D view; checkCIF report

## Figures and Tables

**Table 1:** Hydrogen-bond geometry (Å, °)

*D*—H⋯*A*	*D*—H	H⋯*A*	*D*⋯*A*	*D*—H⋯*A*
O1—H1⋯O3^i^	0.82	2.12	2.6989 (12)	128
O1—H1⋯O5	0.82	1.94	2.6247 (14)	140
C7—H7⋯O4^ii^	0.93	2.50	3.4018 (16)	163
C8—H8*A*⋯O3^iii^	0.96	2.60	3.4733 (18)	152
C8—H8*B*⋯O4^iv^	0.96	2.58	3.476 (2)	156

Symmetry codes: (i) 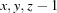; (ii) 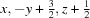; (iii) 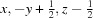; (iv) 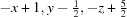.

**Table 2:** Chemical shifts of protons (DMSO-*d*_6_) of 4-hy­droxy-3-meth­oxy-5-nitro­benzaldehyde (**I**)

Chemical shift (*δ*, p.p.m.)	Multiplicity	Number of protons	Assignment
3.962	*s*	3	H9
7.622–7.626	*d*	1	H3
8.095–8.098	*d*	1	H5
9.867	*s*	1	H10

**Table 3:** Chemical shifts of carbons (DMSO-*d*_6_) of 4-hy­droxy-3-meth­oxy-5-nitro­benzaldehyde (**I**)

Chemical shift (*δ*, p.p.m.)	Number of carbons	Assignment
56.78	1	C9
112.52	1	C3
120.87	1	C5
126.81	1	C4
137.04	1	C6
147.73	1	C1
150.03	1	C2
190.42	1	C10

**Table 4:** Experimental details

Crystal data	
Chemical formula	C_8_H_7_NO_5_
*M*_r_	197.15
Crystal system, space group	Monoclinic, *P*2_1_/*c*
Temperature (K)	293
*a*, *b*, *c* (Å)	6.8249 (2), 14.3395 (5), 8.9089 (3)
β (°)	106.678 (4)
*V* (Å^3^)	835.21 (5)
*Z*	4
Radiation type	Cu *K*α
μ (mm^−1^)	1.16
Crystal size (mm)	0.30 × 0.15 × 0.13

Data collection	
Diffractometer	Rigaku Xcalibur Ruby Nova
Absorption correction	Multi-scan (*CrysAlis PRO*; Rigaku OD, 2018 ▸)
*T*_min_, *T*_max_	0.647, 1
No. of measured, independent and observed [*I* > 2σ(*I*)] reflections	3633, 1697, 1574
*R*_int_	0.016
(sin θ/λ)_max_ (Å^−1^)	0.629

Refinement	
*R*[*F* ^2^ > 2σ(*F* ^2^)], *wR*(*F* ^2^), *S*	0.040, 0.174, 0.81
No. of reflections	1697
No. of parameters	127
H-atom treatment	H-atom parameters constrained
Δρ_max_, Δρ_min_ (e Å^−3^)	0.27, −0.17

Computer programs: *CrysAlis PRO* (Rigaku OD, 2018 ▸), *SHELXS97* (Sheldrick, 2008 ▸), *Mercury* (Macrae *et al.*, 2008 ▸), *SHELXL2017* (Sheldrick, 2015 ▸), *PLATON* (Spek, 2020 ▸) and *publCIF* (Westrip, 2010 ▸).

## supplementary crystallographic information

### Crystal data

**Table:**

C_8_H_7_NO_5_	*F*(000) = 408
*M**_r_* = 197.15	*D*_x_ = 1.568 Mg m^−^^3^
Monoclinic, *P*2_1_/*c*	Cu *K*α radiation, λ = 1.54184 Å
Hall symbol: -P 2ybc	Cell parameters from 2167 reflections
*a* = 6.8249 (2) Å	θ = 3.2–75.6°
*b* = 14.3395 (5) Å	µ = 1.16 mm^−^^1^
*c* = 8.9089 (3) Å	*T* = 293 K
β = 106.678 (4)°	Block, yellow
*V* = 835.21 (5) Å^3^	0.30 × 0.15 × 0.13 mm
*Z* = 4	

### Data collection

**Table:**

Rigaku Xcalibur Ruby Nova diffractometer	1697 independent reflections
Radiation source: micro-focus sealed X-ray tube	1574 reflections with *I* > 2σ(*I*)
Mirror monochromator	*R*_int_ = 0.016
Detector resolution: 10.4323 pixels mm^-1^	θ_max_ = 76.0°, θ_min_ = 6.8°
ω scans	*h* = −8→8
Absorption correction: multi-scan (CrysAlis PRO; Rigaku OD, 2018)	*k* = −16→17
*T*_min_ = 0.647, *T*_max_ = 1	*l* = −7→11
3633 measured reflections	

### Refinement

**Table:**

Refinement on *F*^2^	Primary atom site location: structure-invariant direct methods
Least-squares matrix: full	Secondary atom site location: difference Fourier map
*R*[*F*^2^ > 2σ(*F*^2^)] = 0.040	Hydrogen site location: inferred from neighbouring sites
*wR*(*F*^2^) = 0.174	H-atom parameters constrained
*S* = 0.81	*w* = 1/[σ^2^(*F*_o_^2^) + (0.2*P*)^2^] where *P* = (*F*_o_^2^ + 2*F*_c_^2^)/3
1697 reflections	(Δ/σ)_max_ = 0.001
127 parameters	Δρ_max_ = 0.27 e Å^−^^3^
0 restraints	Δρ_min_ = −0.16 e Å^−^^3^

### Special details

**Table:**

Geometry. All esds (except the esd in the dihedral angle between two l.s. planes) are estimated using the full covariance matrix. The cell esds are taken into account individually in the estimation of esds in distances, angles and torsion angles; correlations between esds in cell parameters are only used when they are defined by crystal symmetry. An approximate (isotropic) treatment of cell esds is used for estimating esds involving l.s. planes.

### Fractional atomic coordinates and isotropic or equivalent isotropic displacement parameters (Å^2^)

**Table:**

	*x*	*y*	*z*	*U*_iso_*/*U*_eq_	
O1	0.24420 (14)	0.44411 (6)	0.92251 (10)	0.0442 (3)	
H1	0.258885	0.488242	0.868197	0.066*	
O2	0.19059 (16)	0.31818 (6)	1.10856 (11)	0.0506 (3)	
O3	0.23301 (16)	0.48763 (7)	1.62543 (10)	0.0508 (3)	
O4	0.3265 (2)	0.72037 (7)	1.06656 (14)	0.0655 (4)	
O5	0.29317 (17)	0.62262 (8)	0.87895 (11)	0.0547 (3)	
N1	0.30056 (15)	0.64166 (7)	1.01585 (12)	0.0422 (3)	
C1	0.27609 (15)	0.56669 (8)	1.11855 (13)	0.0353 (3)	
C2	0.24838 (14)	0.47461 (7)	1.06403 (13)	0.0347 (3)	
C3	0.22165 (16)	0.40479 (8)	1.17059 (14)	0.0375 (3)	
C4	0.22427 (16)	0.42865 (8)	1.32044 (13)	0.0386 (3)	
H4	0.206582	0.382706	1.389078	0.046*	
C5	0.25347 (16)	0.52194 (8)	1.37123 (13)	0.0377 (3)	
C6	0.27967 (15)	0.59084 (8)	1.27155 (13)	0.0376 (3)	
H6	0.299441	0.652439	1.305155	0.045*	
C7	0.25478 (19)	0.54494 (9)	1.53231 (14)	0.0424 (3)	
H7	0.273358	0.60689	1.564115	0.051*	
C8	0.1668 (2)	0.24460 (10)	1.21046 (17)	0.0577 (4)	
H8A	0.145807	0.186668	1.154094	0.086*	
H8B	0.287788	0.240337	1.297763	0.086*	
H8C	0.05098	0.257453	1.247879	0.086*	

### Atomic displacement parameters (Å^2^)

**Table:**

	*U*^11^	*U*^22^	*U*^33^	*U*^12^	*U*^13^	*U*^23^
O1	0.0671 (5)	0.0406 (5)	0.0279 (5)	−0.0002 (3)	0.0184 (4)	−0.0015 (3)
O2	0.0823 (6)	0.0336 (5)	0.0392 (6)	−0.0058 (4)	0.0229 (5)	−0.0032 (3)
O3	0.0749 (6)	0.0513 (6)	0.0302 (5)	−0.0034 (4)	0.0214 (4)	−0.0005 (3)
O4	0.1106 (9)	0.0351 (6)	0.0529 (7)	−0.0071 (5)	0.0268 (6)	0.0028 (4)
O5	0.0838 (7)	0.0512 (6)	0.0326 (6)	−0.0033 (4)	0.0223 (5)	0.0063 (4)
N1	0.0540 (5)	0.0378 (6)	0.0353 (6)	−0.0005 (4)	0.0135 (4)	0.0052 (4)
C1	0.0423 (5)	0.0336 (6)	0.0304 (6)	0.0017 (4)	0.0112 (4)	0.0029 (4)
C2	0.0415 (5)	0.0366 (6)	0.0274 (6)	0.0017 (4)	0.0120 (4)	0.0014 (4)
C3	0.0481 (5)	0.0335 (7)	0.0318 (6)	−0.0002 (4)	0.0126 (4)	0.0002 (4)
C4	0.0517 (6)	0.0353 (6)	0.0316 (6)	−0.0014 (4)	0.0163 (5)	0.0022 (4)
C5	0.0453 (6)	0.0396 (7)	0.0296 (6)	0.0012 (4)	0.0131 (4)	−0.0015 (4)
C6	0.0469 (6)	0.0340 (6)	0.0325 (7)	0.0010 (4)	0.0124 (5)	−0.0017 (4)
C7	0.0575 (6)	0.0407 (7)	0.0312 (6)	0.0001 (4)	0.0162 (5)	−0.0024 (4)
C8	0.0881 (9)	0.0352 (7)	0.0513 (8)	−0.0040 (6)	0.0225 (7)	0.0040 (5)

### Geometric parameters (Å, º)

**Table:**

O1—C2	1.3271 (14)	C3—C4	1.3733 (16)
O1—H1	0.82	C4—C5	1.4080 (17)
O2—C3	1.3510 (15)	C4—H4	0.93
O2—C8	1.4310 (15)	C5—C6	1.3740 (15)
O3—C7	1.2068 (16)	C5—C7	1.4700 (16)
O4—N1	1.2097 (15)	C6—H6	0.93
O5—N1	1.2367 (15)	C7—H7	0.93
N1—C1	1.4520 (15)	C8—H8A	0.96
C1—C6	1.3998 (15)	C8—H8B	0.96
C1—C2	1.4008 (17)	C8—H8C	0.96
C2—C3	1.4270 (15)		
			
C2—O1—H1	109.5	C5—C4—H4	119.7
C3—O2—C8	116.83 (9)	C6—C5—C4	120.54 (10)
O4—N1—O5	122.31 (11)	C6—C5—C7	120.27 (11)
O4—N1—C1	119.11 (11)	C4—C5—C7	119.18 (11)
O5—N1—C1	118.58 (10)	C5—C6—C1	118.84 (11)
C6—C1—C2	122.25 (11)	C5—C6—H6	120.6
C6—C1—N1	117.22 (11)	C1—C6—H6	120.6
C2—C1—N1	120.52 (10)	O3—C7—C5	123.43 (12)
O1—C2—C1	127.16 (10)	O3—C7—H7	118.3
O1—C2—C3	115.39 (10)	C5—C7—H7	118.3
C1—C2—C3	117.46 (10)	O2—C8—H8A	109.5
O2—C3—C4	125.69 (11)	O2—C8—H8B	109.5
O2—C3—C2	114.08 (10)	H8A—C8—H8B	109.5
C4—C3—C2	120.22 (11)	O2—C8—H8C	109.5
C3—C4—C5	120.69 (11)	H8A—C8—H8C	109.5
C3—C4—H4	119.7	H8B—C8—H8C	109.5
			
O4—N1—C1—C6	1.36 (17)	O1—C2—C3—C4	179.72 (9)
O5—N1—C1—C6	−178.32 (10)	C1—C2—C3—C4	−0.22 (16)
O4—N1—C1—C2	−179.20 (11)	O2—C3—C4—C5	−178.50 (10)
O5—N1—C1—C2	1.12 (16)	C2—C3—C4—C5	−0.07 (17)
C6—C1—C2—O1	−179.40 (10)	C3—C4—C5—C6	0.07 (17)
N1—C1—C2—O1	1.19 (17)	C3—C4—C5—C7	179.82 (10)
C6—C1—C2—C3	0.52 (16)	C4—C5—C6—C1	0.22 (16)
N1—C1—C2—C3	−178.89 (8)	C7—C5—C6—C1	−179.52 (10)
C8—O2—C3—C4	−2.73 (18)	C2—C1—C6—C5	−0.53 (16)
C8—O2—C3—C2	178.76 (10)	N1—C1—C6—C5	178.90 (8)
O1—C2—C3—O2	−1.68 (14)	C6—C5—C7—O3	−179.85 (11)
C1—C2—C3—O2	178.39 (9)	C4—C5—C7—O3	0.40 (19)

### Hydrogen-bond geometry (Å, º)

**Table:**

*D*—H···*A*	*D*—H	H···*A*	*D*···*A*	*D*—H···*A*
O1—H1···O3^i^	0.82	2.12	2.6989 (12)	128
O1—H1···O5	0.82	1.94	2.6247 (14)	140
C7—H7···O4^ii^	0.93	2.50	3.4018 (16)	163
C8—H8*A*···O3^iii^	0.96	2.60	3.4733 (18)	152
C8—H8*B*···O4^iv^	0.96	2.58	3.476 (2)	156

Symmetry codes: (i) *x*, *y*, *z*−1; (ii) *x*, −*y*+3/2, *z*+1/2; (iii) *x*, −*y*+1/2, *z*−1/2; (iv) −*x*+1, *y*−1/2, −*z*+5/2.
